# Targeting TREM-1 receptors with metformin and pravastatin modulate monosodium iodoacetate-induced osteoarthritis

**DOI:** 10.1007/s10787-025-01738-6

**Published:** 2025-05-03

**Authors:** Eman R. Al Sawy, Mona M. Saber, Noha N. Nassar, Nesrine S. El Sayed

**Affiliations:** 1 Pharmacology and Toxicology Department, Faculty of Pharmacy, Cairo University, Kasr El-Aini st., Cairo, 11562 Egypt; 2 Pharmacology and Toxicology Department, October University for Modern Science and Arts (MSA), Giza, Egypt

**Keywords:** Osteoarthritis, Metformin, Pravastatin, TREM-1, COMP, PI3K

## Abstract

Osteoarthritis (OA), a degenerative joint condition, affects entire joints, including the tissues around it, causing pain, swelling and stiffness. This study explored the combined therapeutic effects of metformin and pravastatin on knee OA induced by monosodium iodoacetate (MIA) in rats. The study encompassed five animal groups (*n* = 10 per group) which were allocated as follows: group 1 received 100 µL of sterile saline injected intra-articularly into the left knee joint (control group) while groups 2–5 were injected with 2 mg MIA in 100µL normal saline into the intraarticular space. After 2 weeks, group 3 received oral metformin (100 mg/kg) for 2 weeks, group 4 received oral pravastatin (10 mg/kg) for 2 weeks and group 5 received both metformin 100 mg/kg and pravastatin 10 mg/kg orally for 2 weeks. The combination of metformin and pravastatin showed to be the most effective in terms of improving radiologic and histologic findings in knee OA. This combination also reduced both serum C reactive protein (CRP) and cartilage oligomeric matrix protein (COMP), while increasing serum collagen type II (COL2). Combining metformin and pravastatin also reduced AKT1, PI3K, STAT3 and IL-6 in the knee joint homogenate. TREM-1 levels showed the greatest reduction with the combination therapy. Furthermore, improvement was noted in oxidative stress markers with an increase in non-protein thiols and a decrease in malondialdehyde. In conclusion, the current study shows a therapeutic potential effect of combining metformin and pravastatin in OA, which could be partly attributed to TREM-1 signaling pathway.

**Graphical abstract:**

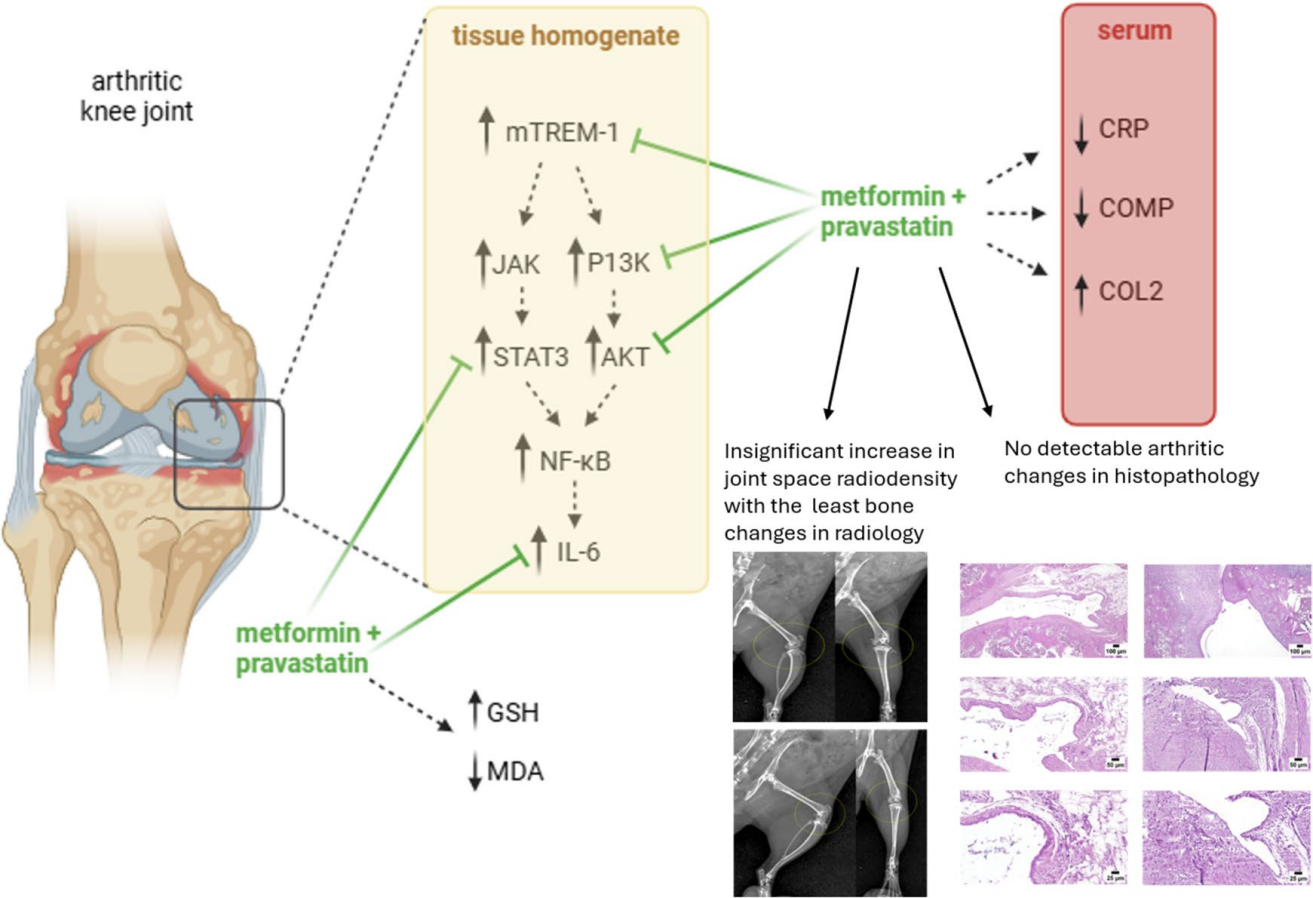

## Introduction

Previously, OA used to be thought of as a mere “wear and tear” condition and was often incorrectly referred to as a degenerative disease that affects joints. Nevertheless, the development of OA is far more intricate than simple wear, encompassing an inflammatory process (Lane et al. 2011; van den Bosch et al. 2020). Numerous elements, such as proteases, pro-inflammatory mediators, and biomechanical variables, are crucial to the pathophysiology of OA.

Research indicates that tissue injury can trigger the innate immune response, which is linked to OA (Liu-Bryan and Terkeltaub 2012; van den Bosch et al. 2020). Noteworthy, following enough matrix damage, the articular cartilage does not show an appropriate reaction for healing thus making the damage irreversible possibly due to its avascular nature. Furthermore, collagen, proteoglycans, fibronectin, cartilage oligomeric protein (COMP), fibromodulin, and other matrix protein fragments are liberated from the broken matrix (Sofat 2009). These fragments trigger the innate immunity response and further encourage the activation of degradative pathways by activating integrins and toll-like receptors (TLRs) (Klesney-Tait et al. 2006; Loeser et al. 2012) further contributing to a feedforward ongoing articular damage.

During tissue damage, when it comes to identifying invasive infections or alerting the host, cells of innate immunity are essential. The function of TLRs, a family of pattern recognition receptors, in innate immunity in both infectious as well as non-infectious disorders is widely recognized. It has been reported that the triggering receptors expressed on myeloid cells (TREMs), a different family of innate immune receptors, interact with TLRs to affect how much of an inflammatory response occurs (Klesney-Tait et al. 2006; Arts et al. 2013). The TREM-family comprises both the TREM-1 and 2 receptors where TREM-1, is known to cause and intensify inflammation when it is activated particularly in concert with TLR signaling. Notably, TREM-1exists in two different forms; a soluble protein and a membrane-bound receptor. Membrane TREM-1 is composed of three distinct domains: a trans-membrane portion, an Ig-like structure that is mostly involved in ligand binding and a cytoplasmic tail that binds to the adaptor molecule tyrosine kinase-binding (TYRO) protein (Colonna 2003). On the other hand, activation of the TREM-2 receptor subtype inhibits the inflammatory response (Sigalov 2022).

Noteworthy, granulocytes and monocytes/macrophages myeloid cells heavily express TREM-1. Moreover, ongoing studies, reveal that TREM-1 is also found on parenchymal cells, including those of the lungs, cornea, stomach, and liver during inflammation (Schmaußer et al. 2008; Chen et al. 2008; Rigo et al. 2012). Following the activation of the membrane-bound TREM-1 by various ligands, including bacterial components and damage-associated molecular patterns (DAMPs), TREM-1 associates with the trans-membrane adaptor protein DAP12.This leads to the phosphorylation of DAP12 and the activation of downstream signaling molecules such as spleen tyrosine kinase (SYK), phospholipase-C-gamma (PLCγ), phosphotylinosital 3 kinase (PI3K), and mitogen-activated protein kinase (MAPK) (Tessarz and Cerwenka 2008).The activation of these signaling molecules cause the actin cytoskeleton to reorganize, Ca2 + to mobilize, and transcription factors like nuclear factor of activated T-cells (NFAT), activator protein 1 (AP1), and nuclear factor kappa B (NF-kB) to become active. These transcription factors then go on to transcribe the genes producing pro-inflammatory cytokines, chemokines, and cell-surface chemicals. The resultant effect is enhanced inflammatory milieu as well as the migration of neutrophils and macrophages to the site of infection or injury.

Interestingly, in inflammatory conditions, research has demonstrated that adenosine monophosphate-activated protein kinase (AMPK) activity can prevent cartilage matrix breakdown. Noteworthy, in OA, AMPK activity is generally decreased. This reduction in AMPK activity is associated with several pathologic changes in OA, including impaired mitochondrial function, increased oxidative stress, and inflammation in joint tissues. These abnormalities contribute to cartilage degradation and joint pain. AMPK deficiency accelerates the development of OA (Terkeltaub et al. 2011; Petursson et al. 2013; Zhou et al. 2017), as demonstrated in a number of inflammatory conditions.

This finding greatly corroborates the utility of metformin in OA due to its ability to activate the nutrient sensor 5′ adenosine monophosphate-activated protein kinase (AMPK). Of note, Nerstedt et al. highlighted the possible role of AMPK in inhibiting STAT3, using metformin, and hence regulating the inflammatory response induced by the cytokine IL-6 in human liver cells (Nerstedt et al. 2010).

Several studies highlighted the beneficial effect of metformin in preventing OA development (Baker et al. 2023; Wang et al. 2024). Other studies explored its role in the treatment of the disease. When individuals who have both type II diabetes and OA used either a combination of metformin plus COX-2 inhibitors versus inhibitors of COX-2 alone, the joint replacement rate was lower with the combination than with COX-2 inhibitors alone. Likewise, patients having radiographic evidence of knee OA along with obesity who took metformin showed less cartilage volume loss than those who did not (Lu et al. 2018; Wang et al. 2019). Metformin, primarily recognized for its ability to reduce blood sugar levels by activating AMPK, also demonstrated additional potential in slowing the advancement of osteoarthritis. This is attributed to its anti-inflammatory characteristics and its possible role in maintaining cartilage health (Halabitska et al. 2024).

Likewise, the utility of statins in management of OA has proposed through as inhibiting the HMG-CoA reductase and impacting the mevalonate pathway. Pahan et al. demonstrated that mevalonate metabolites were involved in the induction of inflammatory mediators and that HMG-CoA reductase inhibitors reduced the induction of inflammatory mediators (IL-6, IL-1β, TNF-α and iNOS) (Pahan et al. 1997). Noteworthy, cell culture studies of the chondrocytes provide evidence of the statins’ potential as anti- inflammatory agents in a dose-dependent manner, an effect that was reversed by the addition of mevalonate confirming the implication of the HMG-CoA reductase activity. In addition, a reduction was noted in the level of inflammatory cells found in the synovial membrane and reduced monocyte chemotactic protein-1 (MCP-1) expression by the synovial tissue. Furthermore, some studies reported that statins can inhibit the activity of TREM-1 and its myeloid cells expression (Liu et al. 2017; Wang et al. 2018; Dai et al. 2019). According to one study, pravastatin prevented peripheral blood mononuclear cells (PBMCs) activated with lipopolysaccharide from expressing TREM-1 and releasing sTREM-1 (Dai et al. 2019). In addition, it decreased TNF-α and IL-6 production in supernatants of cell culture. The authors concluded that such effects could be attributed to interference in NF-κB signaling. Pravastatin improved hyperlipidemic mice’s atherosclerosis by decreasing inflammatory cells in atherosclerotic plaques along with lipid deposits (Wang et al. 2018). Furthermore, TNF-α, IL-1, DAP12 and TREM-1 expression were all reduced with pravastatin administration.

Despite the presence of recent reports that document the positive effect of metformin or statin on OA, no previous studies explored the consequence of combining metformin and statins on the disease. To this end, the present study was carried out to explore the potential therapeutic effects of combining metformin and pravastatin on knee OA through modulating TREM-1 signaling pathway.

## Material and methods

### Drugs and chemicals

Monosodium iodoacetate (MIA) was obtained from Sigma-Aldrich, Inc. (MO, USA) while metformin and pravastatin, were obtained from CID Co. (Cairo, Egypt) and HI PHARM (Cairo, Egypt), respectively. Both drugs were supplied in a powder form and normal saline was used as a solvent. Thiopental used for anesthesia was supplied by EIPICO (Tenth of Ramadan City, Egypt).

### Establishment of a rat OA model

The Institutional Animal Care and Use Committee of Cairo University (Permit number: PT3026) accepted the study, which followed the Guide for Care and Use of Laboratory Animals provided by the US National Institutes of Health (NIH Publication No. 85-23, revised 2011). In-house bred adult 50 male albino Sprague Dawley rats weighing 150–200 g from the Faculty of Pharmacy, Cairo University animal house were used. Within the animal facility associated with the Faculty of Pharmacy, rats were housed under constant temperature (~ 20–23 °C) and humidity (~ 50%) with a 12h light/dark cycle. They had unrestricted access to water and were given standard rodent chow for food.

They were randomly allocated into five groups each composed of ten rats. After anesthesia with thiopental, the control group received 50 μl of sterile saline via intraarticular injection into the knee joint. The other groups were given 2 mg of MIA in 50-µl normal saline by injection into the intraarticular knee joint space to induce OA. After 14 days of MIA injection, one group served as a positive control group, a second group received metformin 100-mg/kg orally for 14 days (Na et al. 2021), a third group received pravastatin 10-mg/kg/day orally for 14 days (Liang et al. 2022) and a fourth group received both 100 mg/kg of metformin and 10 mg/kg of pravastatin orally for 14 days.

### Radiological assessment

After anesthesia using ketamine (50 mg/kg)/xylazine (2 mg/kg) mixture, the hind limbs of each rat were straightened by an adhesive tape (Saber et al. 2023). Fisher^®^ X-ray device (Fisher R183, Emerald tube 125) was used for imaging the left stifle joint at the lateromedial and craniocaudal views.

### Enzyme-linked immunosorbent assay (ELISA)

Serum samples preparation involved allowing samples to clot at room temperature for 20 min before being centrifugated for 10 min at approximately 3000 rpm. The serum samples were then stored for later use at − 80 °C. To thoroughly remove excess blood, ice-cold PBS was used to rinse the tissue collected after being minced into small pieces. Using a glass homogenizer on ice, tissue pieces were homogenized in PBS after being weighed. The homogenates were then centrifuged for 5 min at 5000 × g to get the supernatant. The serum/ tissue homogenate samples or standards were added to the microplate that has been coated with an antibody specific to the protein of interest. Standards or samples, a biotin-conjugated detection antibody and an avidin-horseradish peroxidase (HRP) conjugate were then added sequentially with washing excess away. Next, a substrate solution was added that reacts with the HRP enzyme producing a change in color or light development. At a wavelength of 450 nm ± 10 nm, a microplate reader was used to measure the optical density.

Collagen Type II (COL2) (Cat. # MBS2702065, MyBioSource, Inc., CA, USA), Cartilage oligomeric matrix protein (COMP) (Cat. # MBS267386, MyBioSource, Inc., CA, USA) and C reactive protein (CRP) (Cat. # SCA821Ra, CLOUD-CLONE CORP., TX, USA) were detected in serum samples using ELISA kits as per the instructions of the manufacturer. Thermo Scientific Pierce BCA Protein Assay Kit (Cat. # 23,225 and 23,227, Thermo Fisher Scientific Inc., Massachusetts, USA) was used to detect and quantify total protein colorimetrically. Protein kinase B (AKT1) (Cat. # LS-F49321, LifeSpan BioSciences, Inc., MA, USA), phosphotylinosital 3 kinase (PI3K) (Cat. # MBS260381, MyBioSource, Inc., CA, USA), signal transducer and activator of transcription 3 (STAT3) (Cat. # MBS2515874, MyBioSource, Inc., CA, USA) and interleukin-6 (IL-6) (Cat. # SEA079Ra, CLOUD-CLONE CORP., TX, USA) were detected in tissue homogenates using ELISA kits as per the instructions of the manufacturer.

### Quantitative real-time polymerase chain reaction (qRT-PCR)

A Total RNA isolation kit (GeneDireX, Inc., Taoyuan City, Taiwan) was used to extract total RNA form the frozen serum samples according to the protocol of the manufacturer. Yield and quality of extracted RNA were assessed using the Qubit™ RNA HS Assay kit (Invitrogen Inc., CA, USA). cDNA was synthesized using 1 µg of total RNA using the SuperScript VILO cDNA synthesis kit (Cat. # 11,754–050, Life Technologies, CA, USA) as per the manufacturer’s instructions. Using three technical replicates, qPCR assay was carried out. Using SYBR green PCR master mix (Qiagen), amplification was done in a total volume 20 μl as follows: 10-μl 2X SYBR mix, 10-ng cDNA and 300 nM of forward and reverse primers for the targets genes (TREM-1) (Forward AGGAAGGCTTGGCAGAGGC and Reverse ACAGGGTCGTTCGGAGGAT). Relative transcript levels of TREM-1 were calculated according to 2-ΔΔct method (Schmittgen and Livak 2008) with β-Actin used as a reference gene (Forward CTATCGGCAATGAGCGGTTCC and Reverse TGTGTTGGCATAGAGGTCTTTACG). We repeated the experiments three times, and the three independent biologic replicates were used to calculate the mean values and standard errors. Using paired student’s t test, the significance of differences between data sets was evaluated.

### Colorimetric assay

In tissue homogenates, the oxidative stress markers thiobarbituric acid derivative (TBARS) measured as malondialdehyde (MDA) and non-protein thiols (NPSH) were estimated (Bio-diagnostic, Giza, Egypt). After tissue homogenization and centrifugation, the supernatant was removed for assay and stored on ice. MDA, a lipid peroxidation measurement, was estimated based on its reaction in an acidic medium with thiobarbituric acid at 95℃ for 30 min to produce a resultant pink product, the absorbance of which can be measured at 534 nm. NPSH were estimated based on the reduction of 5,5′ dithiobis (2-nitrobenzoic acid) to form a yellow product whose absorbance, when measured at 405 nm, is directly proportional to NPSH concentration.

### Histopathological examination

Knee joints were collected, at the end of the experiment, from each group. Ten% formalin solution and decalcifying solution-lite (Sigma-Aldrich Inc., MO, USA) were used for fixing and decalcifying the tissues, respectively. The tissues were embedded in paraffin. After being cut, xylene was used to dewax 4- to 5-μm thick sections which were dehydrated through an alcohol gradient. Finally, hematoxylin and eosin (H&E) and safranin O were used to stain these sections. Semiquantitative grading was used to score histopathological changes. The grading included five scores as follows: 4, severe synovitis and erosion with loss of normal joint architecture; 3, moderate synovitis and erosion with a change in joint architecture; 2, synovitis with some marginal erosion but with maintained joint architecture; 1, minimal synovitis without cartilage/bone erosion and 0, normal.

### Statistical analysis

Data are presented as means ± SD. GraphPad Prism 9.0.0 software was used to perform statistical analysis. One-way analysis of variance (ANOVA) was used to perform statistical comparisons, and, for all tests, the statistical significance level was set as *p* < 0.05.

## Results

Table 1 represents radiographic scores assigned to each group based on joint space radiodensity, osteophytic reactivity and bone changes. Joint space radiodensity showed the lowest score in both the control and the combination (metformin + pravastatin) groups with the highest score seen in the MIA group. Similarly, osteophytic reactivity and bone changes were absent in the control group, extreme in the MIA group, and showed the lowest score with the combination treatment. The pravastatin group showed overall lower scores than the metformin group.

**Table 1:** Radiographic score of the injected stifle joint in both lateral and craniocaudal views in the different experimental groups

Group	Joint space radiodensity	Osteophytic reactivity	Bone changes
Control	Very low ( ±)	Not present (−)	Not present (−)
MIA	Extreme (+ + + +)	Extreme (+ + + +)	Extreme (+ + + +)
Metformin	High (+ +)	Moderate (+ +)	Moderate (+ +)
Pravastatin	Low ( +)	Low ( +)	Low ( +)
Metformin + pravastatin	Very low ( ±)	Low ( +)	Very low ( ±)

*MIA* monosodium iodoacetate

### Radiographic assessment

**Fig. 1:**
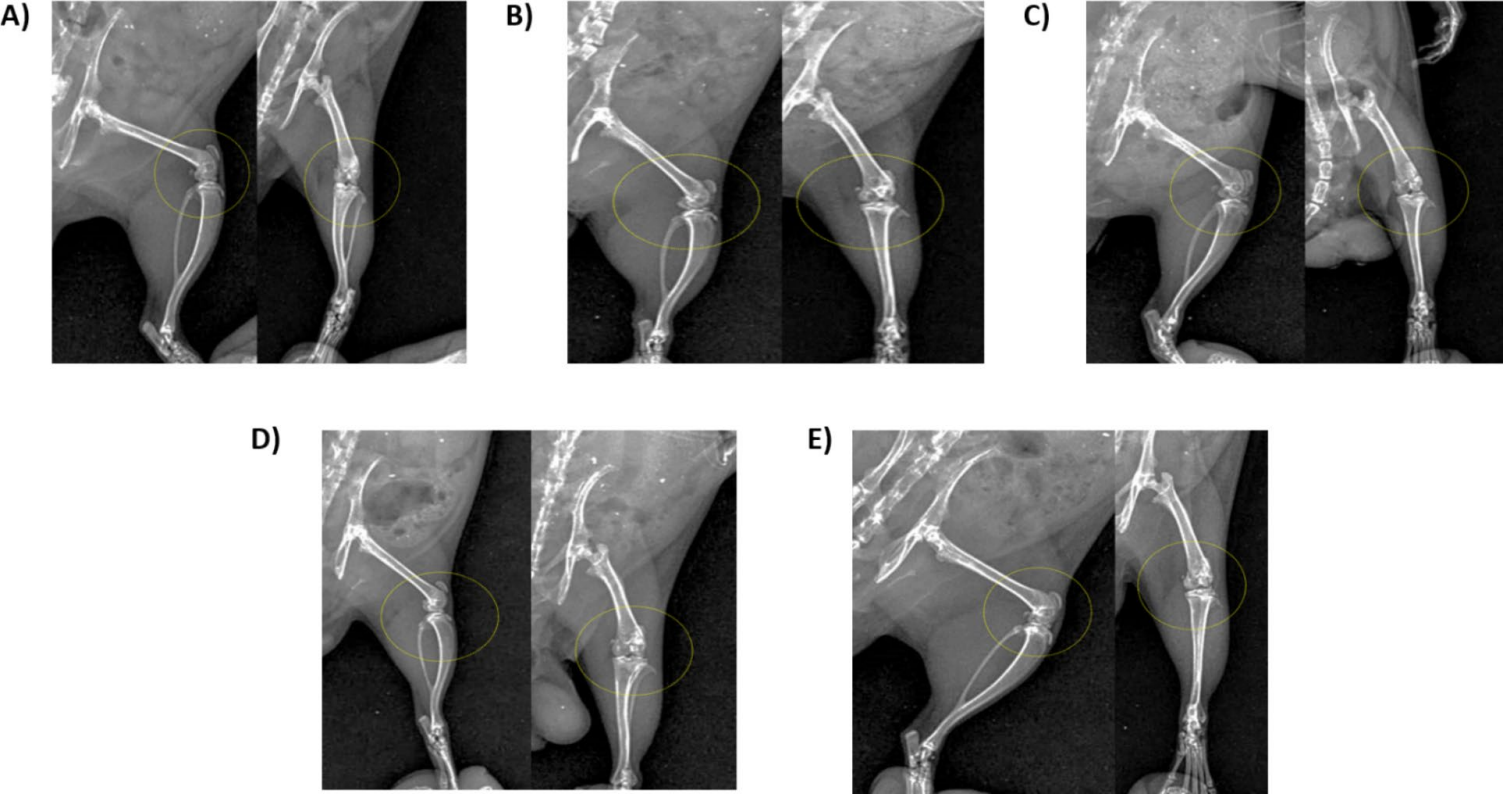
Lateromedial (left) and Craniocaudal (right) radiographic views of the left stifle joint in **A** control group, **B** MIA group, **C** metformin group, **D** pravastatin and **E** metformin and pravastatin group

Radiologically the control group showed slight joint effusion, neither reduction nor increase were seen in joint space radiodensity and the femoral condyles and tibia showed no radiographic abnormalities; Fig. 1A.

MIA injection showed joint capsule effusion and thickening with narrowing and increase in joint space radiodensity. The femoral condyles and tibial surface showed obvious osteo-reactivity and articular surface radiographic changes; Fig. 1B.

Metformin treatment showed slight joint capsule radiographic changes with moderate increase in joint space radiodensity, moderate narrowing and slight to moderate osteo-reactivity appearing at articular surfaces of femur and tibia in anteroposterior (AP) view; Fig. 1C. Pravastatin treatment showed joint capsule effusion with slight increase in joint space radiodensity, scanty to slight osteo-reactivity appeared at the articular surfaces in AP view, no condylar changes; Fig. 1D. Metformin and pravastatin treatment showed slight joint capsule effusion with insignificant increase in joint space radiodensity, neither osteo-reactivity nor condylar changes appeared at the articular surfaces in AP view; Fig. 1E.

### Metformin and pravastatin treatment resulted in a reduction in TREM-1

**Fig. 2:**
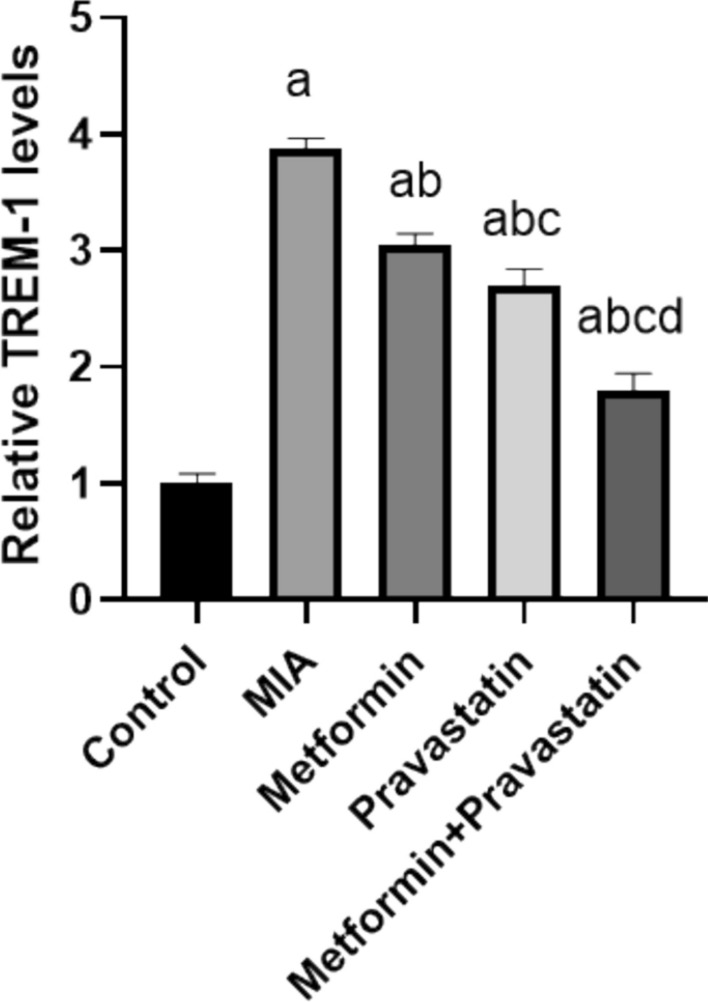
Relative TREM-1 levels in knee joint homogenate among the different groups. One-way ANOVA was used, and Tukey’s multiple comparisons test followed for significance. **a** significantly different from control, **b** significantly different from MIA, **c** significantly different from metformin, and d significantly different from pravastatin (*p* < 0.05)

Figure 2 shows a 2.9-fold increase in TREM-1 level after MIA injection, in comparison to the control group. Metformin and pravastatin treatments decreased TREM-1 levels by 0.8- and 1.2-fold, respectively, compared to the MIA group. Combining both metformin and pravastatin resulted in a twofold decrease in TREM-1 levels.

### Reduction in STAT3 and IL-6 after treatment with metformin and pravastatin

**Fig. 3:**
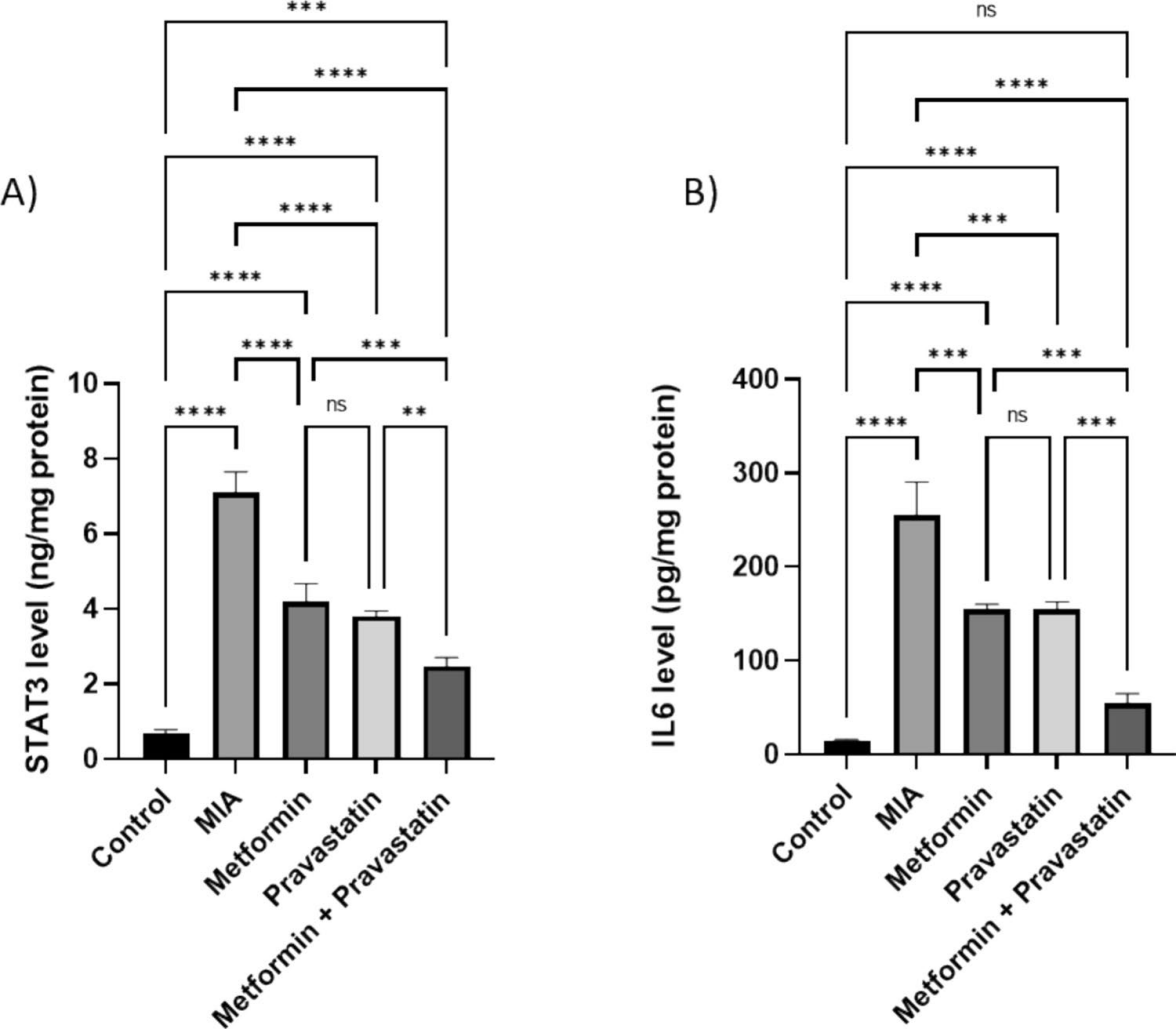
Relative levels of **A** STAT3 and **B** IL6 in the rat knee joint homogenate after treatment with metformin, pravastatin, and metformin and pravastatin combination. One-way ANOVA was used, and Tukey’s multiple comparisons test followed for significance. *p < 0.05, **p < 0.01, ***p < 0.001, ****p < 0.0001

MIA injection increased both STAT3 and IL-6 levels in tissue by 9.6- and 15.9-folds, respectively (Fig. 3). Metformin treatment decreased both STAT3 level by 4.3-folds and IL6 level by 6.6-folds. Pravastatin also decreased STAT3 and IL6 levels by 4.9- and 6.7-folds, respectively. The combination of metformin and pravastatin decreased STAT3 level by 6.9-folds and decreased IL6 level by 13.3 folds.

### Metformin, pravastatin and their combination decreased PI3K and AKT levels

**Fig. 4:**
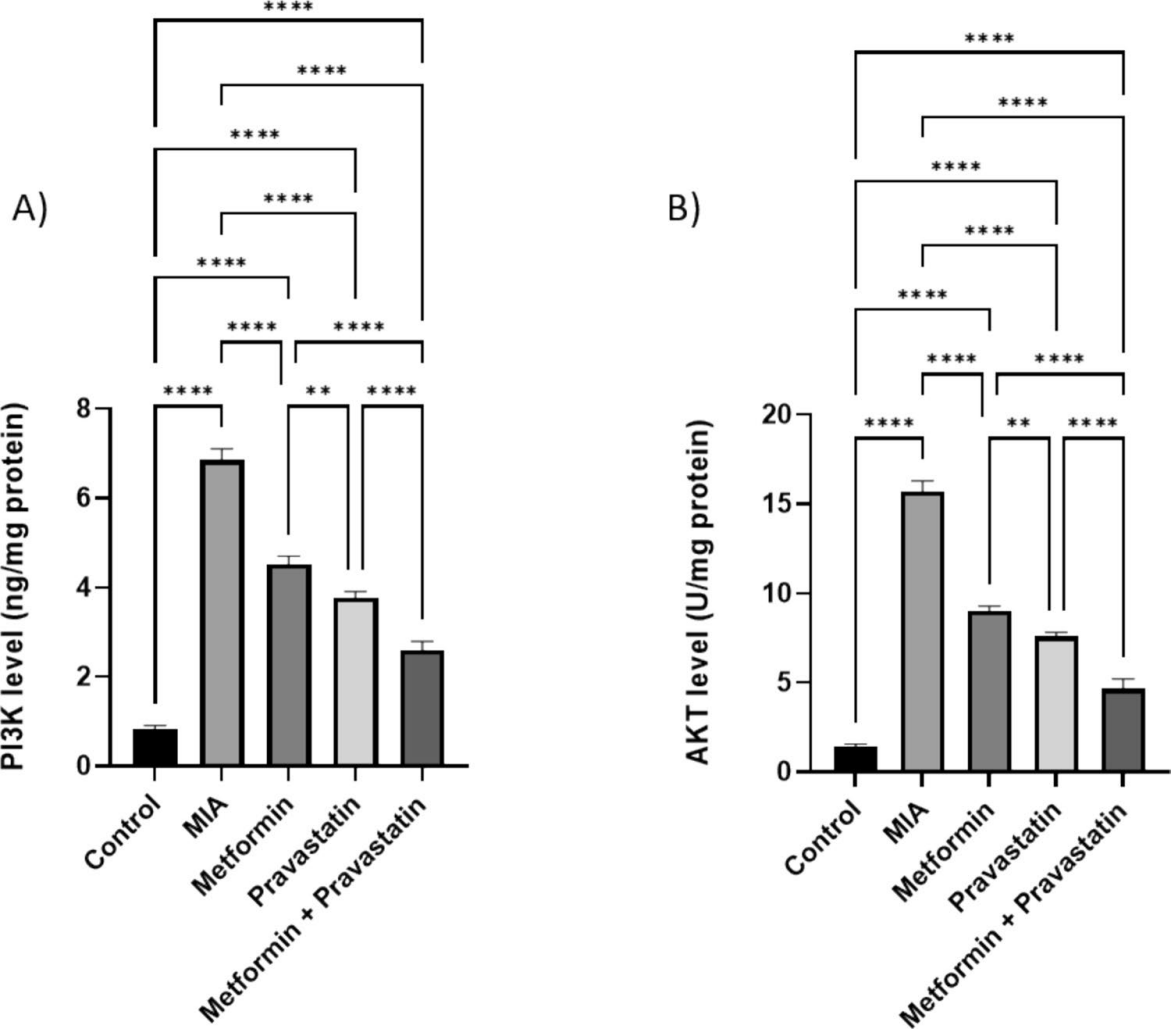
Relative levels of **A** PI3K and **B** AKT in the knee joint homogenate after treating rats with metformin, pravastatin, and a combination of metformin and pravastatin. One-way ANOVA was used, and Tukey’s multiple comparisons test followed for significance. *p < 0.05, **p < 0.01, ***p < 0.001, ****p < 0.0001

After OA induction by MIA, PI3K and AKT levels increased by sevenfolds and tenfolds, respectively (Fig. 4). Metformin administration resulted in a 2.7- and 4.7-folds decrease in PI3K and AKT levels, respectively. Pravastatin administration also decreased PI3K and AKT levels by 3.6- and 5.7-folds, respectively. Administering both metformin and pravastatin resulted in a five- and 7.7-folds decrease in PI3K and AKT levels, respectively.

### Improvement in oxidative stress markers after treatment with metformin and pravastatin

**Fig. 5:**
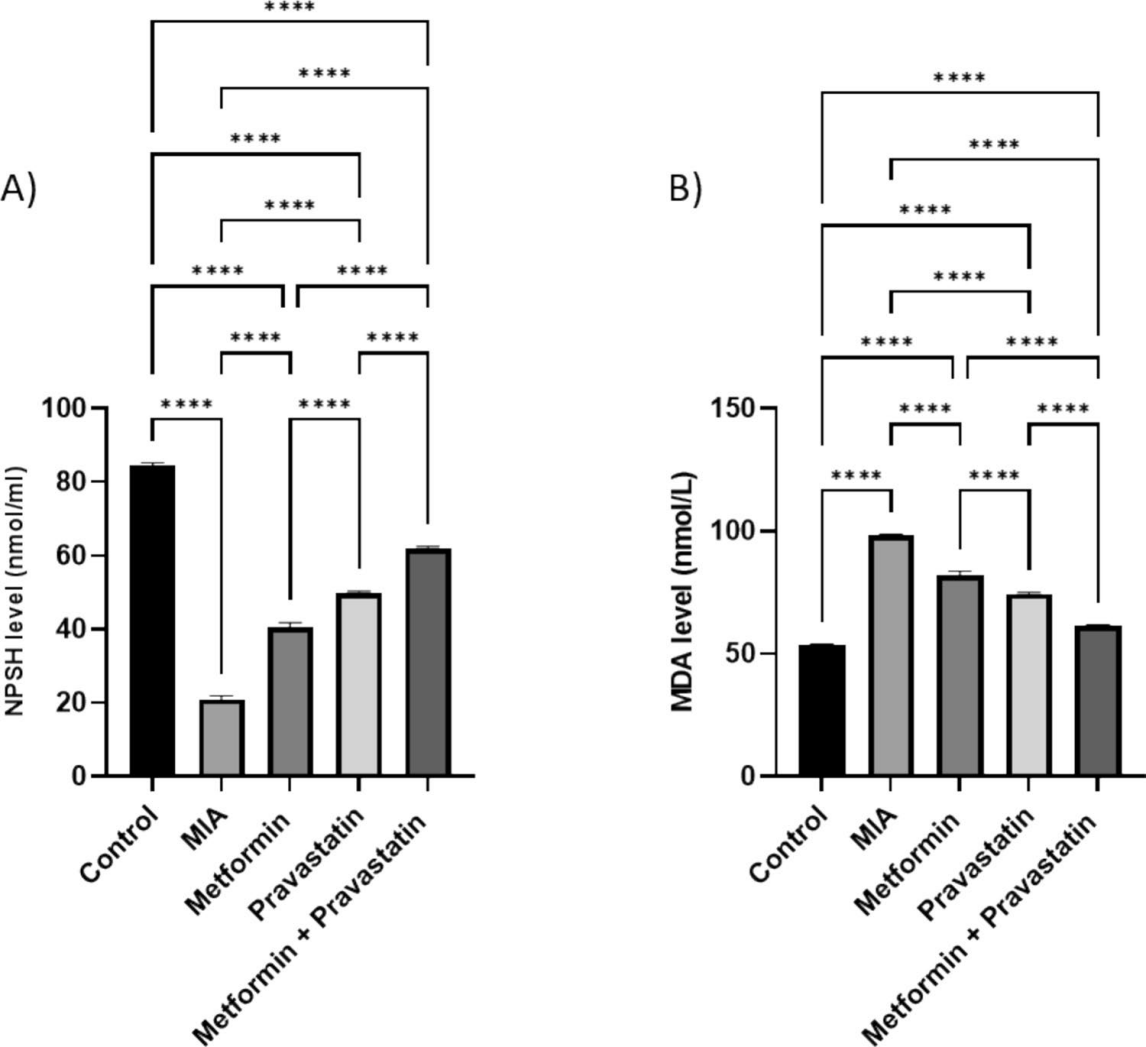
Relative levels of **A** NPSH and **B** MDA in the knee joint homogenate in different treatment groups. One-way ANOVA was used, and Tukey’s multiple comparisons test followed for significance. *p < 0.05, **p < 0.01, ***p < 0.001, ****p < 0.0001

As represented in Fig. 5, injecting MIA decreased NPSH level by 63.4-folds, compared to the control group. Meanwhile, both metformin and pravastatin treatments increased NPSH level by 19.5-and 28.7-folds, respectively, compared to the MIA group. Co-treatment with metformin and pravastatin increased NPSH level by 40.8-folds. Figure 5 also shows a 44.5-fold increase in MDA level after OA induction with MIA. A 16- and a 23.8-fold decrease in MDA was noted after metformin and pravastatin treatments, respectively. Co-treatment with metformin and pravastatin decreased MDA by 36.8-folds.

### Metformin, pravastatin and their combination significantly reduced the knee joint damage.

**Fig. 6:**
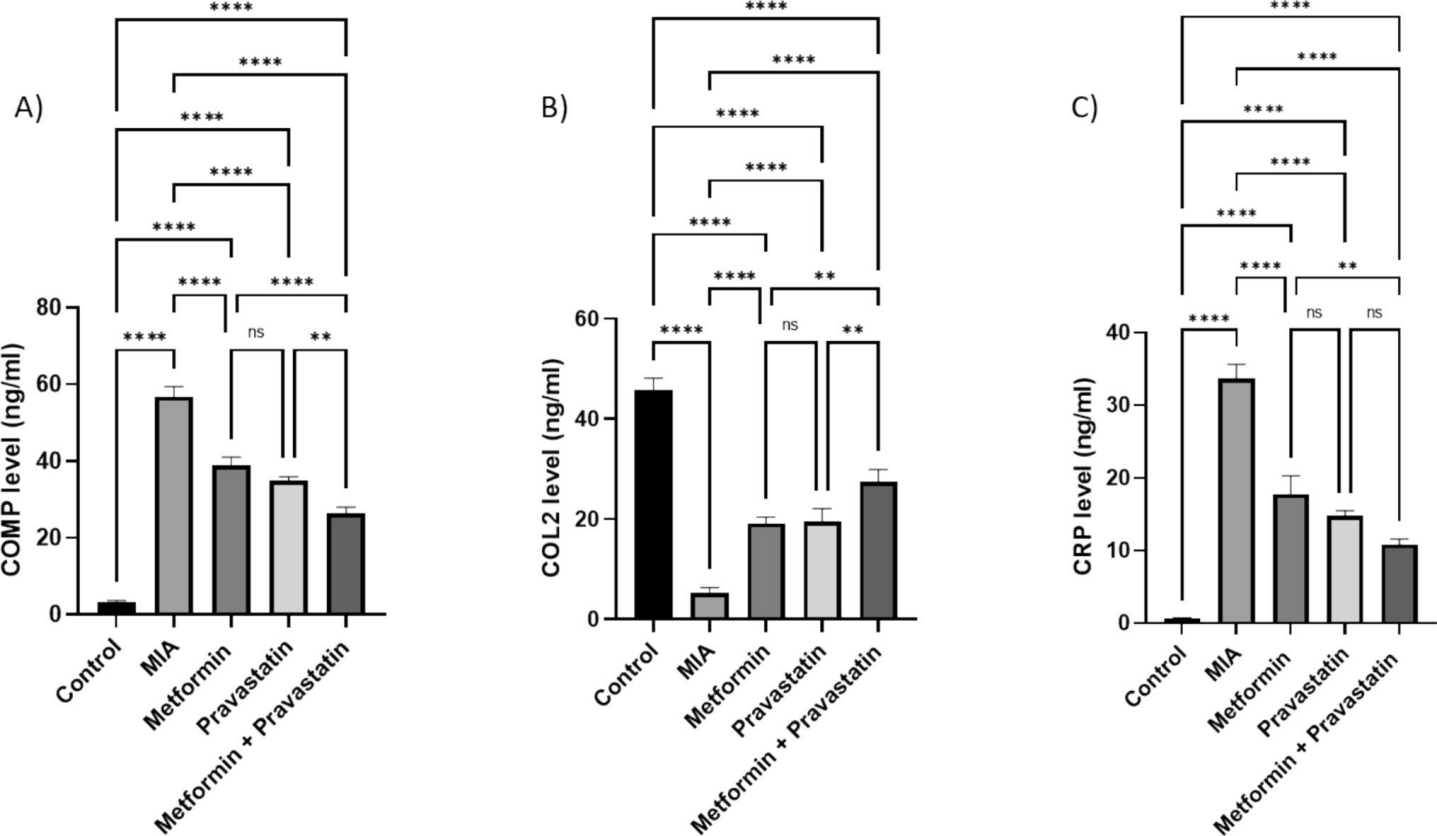
Relative serum levels of **A** COMP, **B** COL2 and **C** CRP in rats treated with metformin, pravastatin, and metformin and pravastatin. One-way ANOVA was used, and Tukey’s multiple comparisons test followed for significance. *p < 0.05, **p < 0.01, ***p < 0.001, ****p < 0.0001

Figure 6 represents relative serum COMP, COL2 and CRP levels among the five groups. Compared to the control group, MIA injection increased COMP and CRP levels by 16.6- and 52.4-folds, respectively, while it decreased COL2 level by 0.88-fold. Treatment with metformin decreased each of the COMP level by 5.6-folds and the CRP level by 25 folds, while it significantly increased COL2 level by 0.3-fold. Pravastatin treatment decreased both COMP and CRP levels by 6.8- and 29.9-folds, respectively, while it significantly increased COL2 level by 0.3-fold. When metformin and pravastatin treatments were combined, both COMP and CRP levels decreased by 9.4- and 36-folds, respectively, while COL2 level increased by 0.49-fold.

### Histopathological findings

**Fig. 7:**
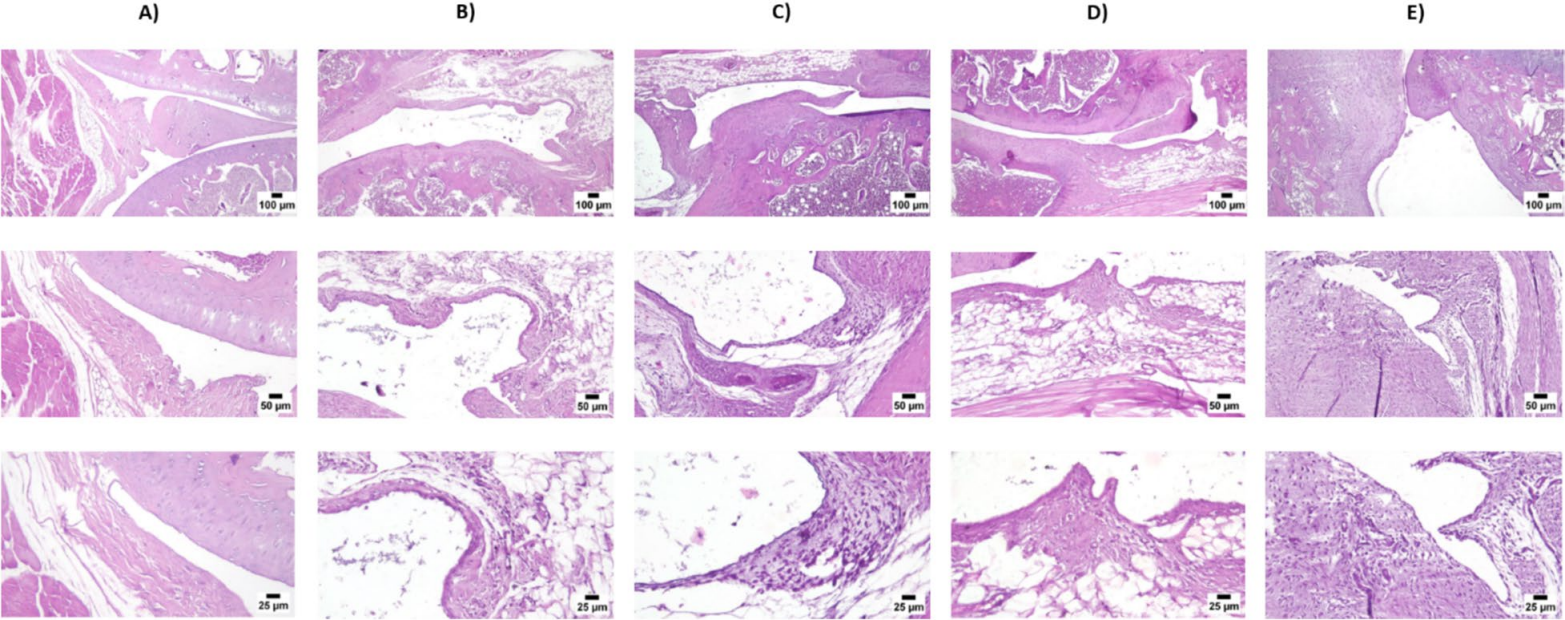
Photomicrographs of **A** control group, **B** MIA group, **C** metformin group, **D** pravastatin group and **E** metformin and pravastatin group. H&E stain (original magnifications × 25, × 50 and × 100)

**Fig. 8:**
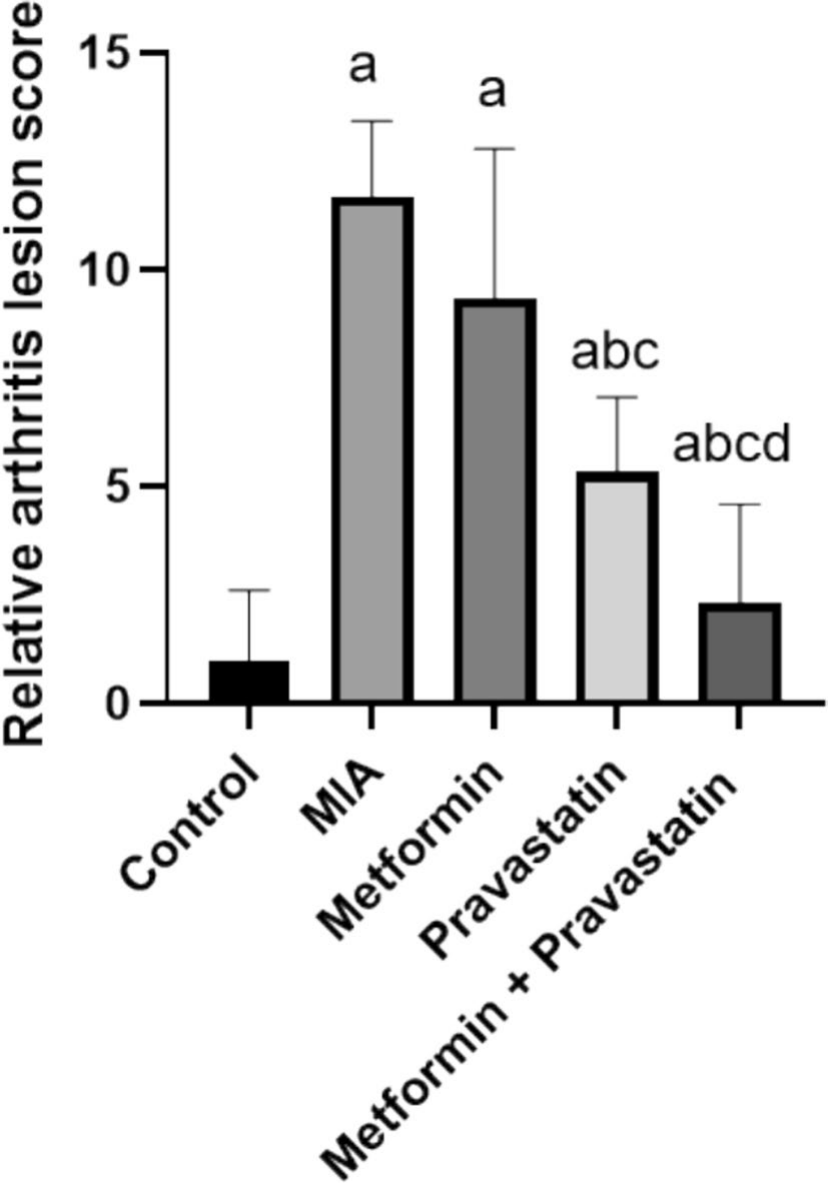
Relative arthritis score between groups. One-way ANOVA was used, and Tukey’s multiple comparisons test followed for significance. **a** significantly different from control, **b** significantly different from MIA, **c** significantly different from metformin, and **d** significantly different from pravastatin (*p* < 0.05)

As shown in Fig. 7, when joint tissue sections from the control group were examined microscopically, they revealed normal structure of the knee joint. Conversely, the MIA group exhibited marked histopathological changes; the periarticular tissue and joint capsule were infiltrated by intense inflammatory cells with marked edema. Synovial lining showed wide areas of sloughed cells. The articular cartilage showed surface damage with the existence of numerous necrosed chondrocytes. Mild improvement was noticed in the metformin-treated group where mild periarticular edema with mononuclear inflammatory cells infiltration were noticed. The articular cartilage was apparently normal. Regarding the pravastatin-treated group, moderate improvement was noticed, only mild periarticular edema was detected with apparently normal articular surfaces. The best protective action was detected in the group that received metformin and pravastatin as all examined joint sections were apparently normal without any detectable changes. Figure 8 represents statistical analysis of histopathological lesion score where it increased by 10.7-folds after MIA injection. Metformin, pravastatin, and their combination decreased lesion score by 2.3-, 6.3- and 9.3-folds, respectively.

## Discussion

The knees, hands, hips, and spine are the most common sites affected by OA causing pain, stiffness and swelling. This study aimed at studying the combined therapeutic effects of metformin and pravastatin in rat OA of the knee joint. The results showed that combining oral metformin and pravastatin for 2 weeks after OA induction was more effective in improving findings associated with OA than using either metformin or pravastatin alone.

Other findings in OA include matrix proteins fragments including COMP which are released from the damaged matrix. In this study, the combination of metformin and pravastatin treatments was found to decrease the levels of COMP and the inflammatory marker CRP in the serum more than treatment with either metformin or pravastatin alone. COL2 levels in serum were also measure and, in agreement with previous studies (Feng et al. 2020; Li et al. 2020a), metformin did increase COL2 expression, but the best results came from combining metformin and pravastatin where a significant increase in COL2 expression was detected.

After mTREM-1 activation, intracellular pathways JAK and PI3K activates, which in turn activates STAT3 and AKT, respectively. These pathways activate transcription factors including NF-κB involved in inflammatory mediators’ production such as IL-6, TNF-α and IL-β (Olivotto et al. 2015). Previous studies of metformin in OA shows its therapeutic potential through AMPK pathway (Feng et al. 2020; Li et al. 2020a, b). Using metformin, a study found that the inflammatory response can be regulated, possibly through AMPK inhibiting STAT3 (Nerstedt et al. 2010). Metformin was found in another study to induce cell cycle arrest in rheumatoid arthritis fibroblast-like synoviocytes, and that this effect could be due to inhibiting IGF-IR/PI3K/AKT/m-TOR pathway (Chen et al. 2019). In the current study, each of PI3K, STAT3 and AKT levels, measured in knee joint homogenate, showed the greatest reduction when metformin and pravastatin treatments were co-administered in comparison to administering either metformin or pravastatin alone. This difference shown with the combination treatment was significant from other treatments.

IL-6, an inflammatory mediator present in osteoarthritic synovial fluid, is involved in OA pathogenesis. Lovastatin treatment was shown to reduce the induction of mediators of inflammation (IL-6, TNF-α, IL-1βand iNOS) (Pahan et al. 1997). Similarly, simvastatin and atorvastatin, dose dependently, has demonstrated a significant inhibition of IL-6 production (Barsante et al. 2005; Dombrecht et al. 2007). In the present study, IL-6 levels were measured in knee joint homogenate, after treatment with metformin in combination with pravastatin. A significant reduction was noted in comparison to each of metformin and pravastatin treatments alone.

Previous studies showed the effect of statins on TREM-1. A study of atherosclerosis in mice showed that pravastatin inhibited TREM-1/DAP12, hence improving atherosclerosis (Wang et al. 2018). In a different study, it was found that TREM-1 mediated inflammation could be inhibited by pravastatin in human PBMCs with NF-κB signaling pathway being involved (Dai et al. 2019). A different study demonstrated that atorvastatin inhibited airway wall remodeling associated with asthma through downregulating the expression of TREM-1 (Liu et al. 2017). However, in this research, the combined effect of metformin and pravastatin on mTREM-1 was studied and, compared to using either metformin or pravastatin alone, it showed a significant decrease in mTREM-1 levels.

MDA results from lipid peroxidation. A previous study reported a significant increase in lipid peroxidation, verified by MDA and 4-hydroxy-2-nonenal production, in human OA synovial cells as compared to those from rheumatoid arthritis patients and controls (Grigolo et al. 2003). On the other hand, GSH, an antioxidant that can prevent lipid peroxidation, was found to improve antioxidant capacity, and modulate pro-inflammatory cytokines expression in human fibroblast-like synoviocytes when hyaluronic acid was supplemented with GSH (Yang et al. 2016). In this study, metformin and pravastatin administration significantly reduced MDA levels in knee joint homogenate when compared to either metformin or pravastatin alone. Meanwhile, non-protein thiol levels significantly increased with the combination treatment as compared to other treatments. Both of which could have a potential role in attenuating the damage associated with OA.

This current study also showed that combining metformin and pravastatin resulted in the least radiographic changes and hardly any detectable changes with apparently normal joint sections in histopathological examination.

To the best of our knowledge, no previous research was performed on the combination of metformin and pravastatin in osteoarthritis. To conclude, this study evaluated the therapeutic effect of combining metformin and pravastatin treatments in OA and the possible involvement of TREM-1 pathway. This combination treatment was found to reduce CRP and COMP in OA knee joints while it increased COL2 expression. The levels of TREM-1, PI3K, AKT, STAT3 and IL-6 were all reduced which goes to suggest a role for TREM-1/DAP12 signaling pathway in developing OA. Radiographic and histologic examination further supported such findings. Future studies are required for further evaluation of the therapeutic role of metformin and pravastatin through TREM-1 pathway.

## Data Availability

Data will be made available on request.
